# Ketogenic Diet and Bipolar Disorder

**DOI:** 10.1192/bjo.2026.11128

**Published:** 2026-06-30

**Authors:** Madhini Sivakumar, Gurpreet Kaler

**Affiliations:** University of Sheffield, Sheffield, United Kingdom

## Abstract

**Aims:**

To understand the influence of adjuvant ketogenic diet on behavioural dysregulation in individuals with bipolar disorder through a review of the available literature.

**Methods:**

A systematic review was conducted across the databases of Medline Ovid, PubMed, PsycINFO, the Cochrane Library, and the University of Sheffield Star Plus library, following the PRISMA guidelines. Relevant articles were identified using Inclusion and Exclusion criteria. Critical appraisal tools (the Critical Appraisal Skills Programme (CASP) and the Joanna Briggs Institute (JBI)) were used to assess the quality of the selected publications.

**Results:**

Twenty-two articles were identified as suitable for the review. Out of this, 10 were systematic reviews, and 6 were case reports. Eighteen publications have deduced that ketogenic diets aid in stabilising mood.

**Conclusion:**

The review identified that ketogenic diets have the potential to reduce behavioural dysregulation in bipolar patients. The descriptive analysis suggests the need for additional clinical research, as the available studies were mainly uncontrolled and included a limited number of participants.

